# Apolipoprotein E genotypes are associated with diabetic peripheral neuropathy in Lebanese adults with type 2 diabetes: a case-control study

**DOI:** 10.3389/fendo.2025.1738873

**Published:** 2025-12-19

**Authors:** Rita Nemr, Sabrina Zidi, Akram Echtay, Eddie Racoubian, Nisrine Beydoun, Wassim Y. Almawi

**Affiliations:** 1 Department of Internal Medicine, Lebanese American University (LAU) Medical Center - Rizk Hospital, Beirut, Lebanon; 2 Group of Mycoplasmas, Laboratory of Molecular Microbiology, Vaccinology, and Biotechnology Development, Pasteur Institute of Tunis, Tunis, Tunisia; 3 Department of Adult Endocrinology and Diabetes, Rafic Hariri University Hospital, Beirut, Lebanon; 4 Department of Laboratory Medicine, St. Marc Medical Center, Beirut, Lebanon; 5 Faculty of Sciences, El-Manar University, Tunis, Tunisia

**Keywords:** alleles, apolipoprotein e, cholesterol, diabetic peripheral neuropathy, genotyping, triglycerides, type 2 diabetes mellitus

## Abstract

**Background:**

Apolipoprotein E (ApoE) affects lipid metabolism and was associated with type 2 diabetes mellitus (T2DM) complications, including diabetic peripheral neuropathy (DPN). Despite improved glycemic control, DPN prevalence continues to rise, indicating mechanisms beyond hyperglycemia. We assessed the association between *APOE* genotypes and DPN susceptibility in patients with T2DM, focusing on dyslipidemia-linked pathways underlying neuropathy susceptibility distinct from glycemic effects.

**Methods:**

The case-control study included 908 Lebanese patients with T2DM (382 with DPN, 526 without) and 695 healthy controls who underwent multimodal DPN assessment (NCS, QST, and MNSI). *APOE* genotyping was performed by PCR-RFLP analysis. Logistic regression models were applied to examine the associations between *APOE* variants and higher odds of DPN.

**Results:**

T2DM patients showed significantly higher frequencies of *ϵ2* and *ϵ4* alleles than controls. Among T2DM patients, those with DPN had significantly higher *ϵ2* allele frequency and lower ϵ3 allele frequency. At the genotype level, *ϵ3*/*ϵ3* genotype demonstrated lower odds of DPN, while *ϵ2/ϵ3*, *ϵ2/ϵ4*, and *ϵ3/ϵ4* were significantly associated with increased odds after adjustment for traditional risk factors. When pooled by allele, *ϵ2*-containing genotypes (*ϵ2/ϵ3* + *ϵ2/ϵ4*; OR (95% CI) = 1.86 [1.38–2.51], and *ϵ4*-containing genotypes (*ϵ3/ϵ4* + *ϵ4/ϵ4* + *ϵ2/ϵ4*; OR (95% CI) = 1.62 [95% CI = 1.08–2.44]) showed high odds of DPN. Lipid profiles varied by genotype: *ϵ4*-containing genotypes displayed atherogenic patterns (elevated total cholesterol and triglycerides, reduced HDL) and were associated with a 1.6-fold higher odds of DPN, while *ϵ2-*containing genotypes showed increased total cholesterol and LDL among DPN patients. Genotype-specific clinical correlations were genotype-specific: *ϵ3*/*ϵ3* was associated with retinopathy and hypertension but protective against nephropathy, while *ϵ3*/*ϵ4* correlated with diabetic complications and dyslipidemia, and *ϵ4*/*ϵ4* linked to a higher BMI.

**Conclusion:**

*APOE* genetic variants, especially *ϵ4*-containing genotypes, are associated with DPN susceptibility among Lebanese T2DM patients, independent of traditional risk factors including glycemic control. These population-specific findings require validation in prospective cohorts before clinical use but indicate potential value for *APOE* genotyping in DPN precision-risk models.

## 1 Introduction

Type 2 diabetes mellitus (T2DM) is a chronic disorder of glucose regulation characterized by insulin resistance and insulin deficiency, contributing significantly to global morbidity and mortality (1, 2). Affecting over 537 million adults worldwide, with a prevalence projected to rise to 783 million by 2045 (3), T2DM is associated with macrovascular and microvascular complications (4, 5). Among these, diabetic neuropathy, particularly peripheral diabetic neuropathy (DPN), represents one of the common and disabling complications, impacting roughly 50% of patients (6, 7). Its effects on sensory function and pain perception significantly reduce quality of life and increase healthcare burdens (7).

DPN involves the progressive loss of somatic and autonomic nerve fibers, typically starting in the extremities, leading to symptoms such as pain and numbness (6, 8). This is compounded by underlying issues such as hyperglycemia, inflammation, vascular dysfunction, and dyslipidemia (9). Despite improved glycemic control, DPN prevalence continues to rise, suggesting mechanisms beyond hyperglycemia alone (6). Although the duration and management of diabetes influence risk (9, 10), persistent variability suggests a significant contribution from genetic factors (11, 12). This is supported by familial clustering (13) and differences observed across ethnic groups (14, 15). Several genetic variants, including those in the aldose reductase, Na/K}-ATPase, and *APOE* genes, have been reported to modulate DPN risk (16, 17).

Apolipoprotein E (ApoE) regulates lipid metabolism, neuronal repair, and inflammation (1, 18). Encoded by the *APOE* gene on chromosome 19q13.2, it has three major alleles defined by rs429358 and rs7412 SNPs: *ϵ2*, *ϵ3*, and *ϵ4*. These form six genotypes (*ϵ2*/*ϵ2*, *ϵ2*/*ϵ3*, *ϵ2*/*ϵ4*, *ϵ3*/*ϵ3*, *ϵ3*/*ϵ4*, and *ϵ4*/*ϵ4*), with distinct receptor-binding and lipid-transport profiles (16, 19), which influence neurobiology (18, 20). While *ϵ3* is metabolically neutral, ϵ4 is linked to hypercholesterolemia and atherosclerosis (21, 22), and ϵ2 is associated with lower LDL-cholesterol, reduced cardiovascular risk (22, 23), and altered lipid profiles affecting DPN susceptibility (24, 25). Although APOE variants are implicated in T2DM complications, data in Middle Eastern populations remain scarce. High consanguinity and unique genetic architecture may modify variant effects. Prior studies across ethnicities report inconsistent *APOE*-DPN associations, ranging from null findings to a fivefold increase in risk. These inconsistencies in these associations likely reflect methodological bias, given that phenotyping varies widely, and as genetic effects differ by ancestry, limiting the generalizability. Small sample sizes (n < 200) further reduce power, highlighting the need for rigorous, ancestry-specific studies with proper covariate control to clarify the role of *APOE* in DPN.

**Figure 1:**
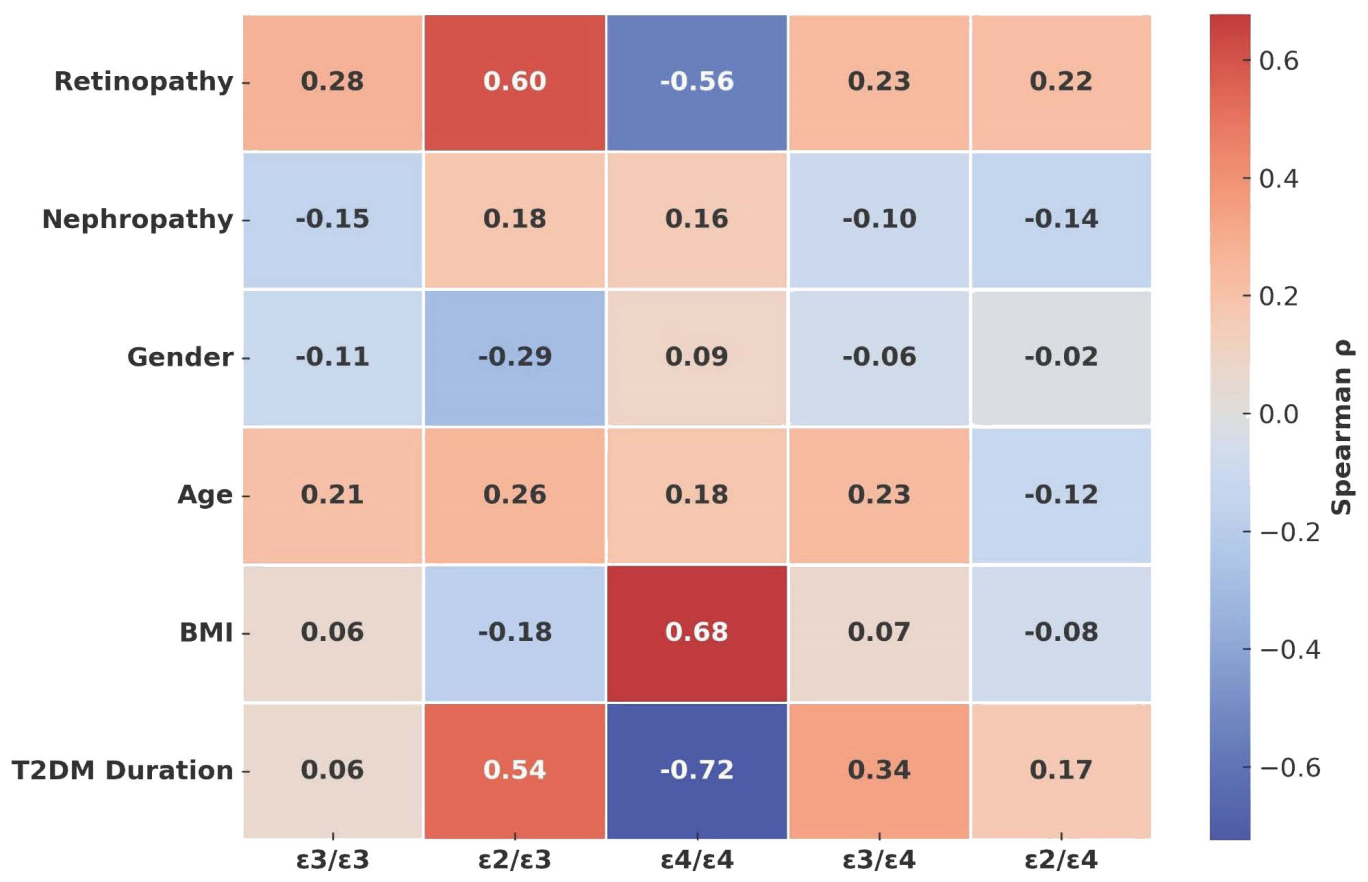
Heatmap showing Spearman correlation coefficients (ρ) between ApoE genotypes and clinical features of DPN. Positive correlations are shown in red, negative in blue, with intensity reflecting effect size. Significant associations were observed for ϵ2/ϵ3 with retinopathy and disease duration, and for ϵ4/ϵ4 with BMI and inverse disease duration.

*APOE* isoforms influence neuronal repair, lipid metabolism, and inflammatory responses, all of which are central to DPN pathogenesis (26). The ϵ4 allele demonstrates reduced antioxidant capacity and altered lipid handling, potentially exacerbating neural injury (27, 28), whereas ϵ2 variants are linked to hypertriglyceridemia, contributing to microvascular dysfunction (29, 30). Given that dyslipidemia is an independent risk factor for DPN, APOE variants likely modulate neuropathy susceptibility through lipid homeostasis mechanisms (31) (detailed mechanistic pathways are illustrated in **Figure 1** and discussed below). Despite comprehensive studies establishing *APOE*’s role in cardiovascular and neurodegenerative conditions (28), its specific contribution to T2DM complications has yielded inconsistent findings. These differences are likely due to ethnic population variations (1, 32), heterogeneous study methodologies, confounding variables, and complex gene-gene interactions that vary across different ancestral backgrounds (32, 33), necessitating more standardized research approaches (34).

The Lebanese population provides an opportunity to study diabetic complications, given its distinct genetic architecture and rising T2DM prevalence marked by urbanization, consanguinity, and admixture (1, 35). *APOE* allele frequencies vary across the Middle Eastern population (36), and existing DPN prediction models lack consensus on genetic risk markers, potentially affecting association power and the transferability of genetic odds estimates across regional subgroups, underscoring the need to investigate the association of DPN modifier genes, including *APOE*, in diverse populations. This study examines APOE alleles/genotypes and higher odds of DPN in Lebanese T2DM patients, focusing on genotype-specific lipid profiles and their link to DPN susceptibility. We assessed whether *APOE* variants are associated with increased odds of DPN after adjustment for clinical and metabolic factors, including glycemic control, lipid profiles, and anthropometric measures. We recruited a well-powered cohort and applied rigorous multimodal neuropathy phenotyping with systematic adjustment for lipid-lowering therapy and key demographic, metabolic, and clinical confounders. We hypothesize that APOE genotypes influence DPN risk via glycemia-independent dyslipidemia pathways, though our cross-sectional design limits inference on temporal links between genotype, lipid alterations, and neuropathy onset.

## 2 Subjects and methods

### 2.1 Study subjects

**Figure 2:**
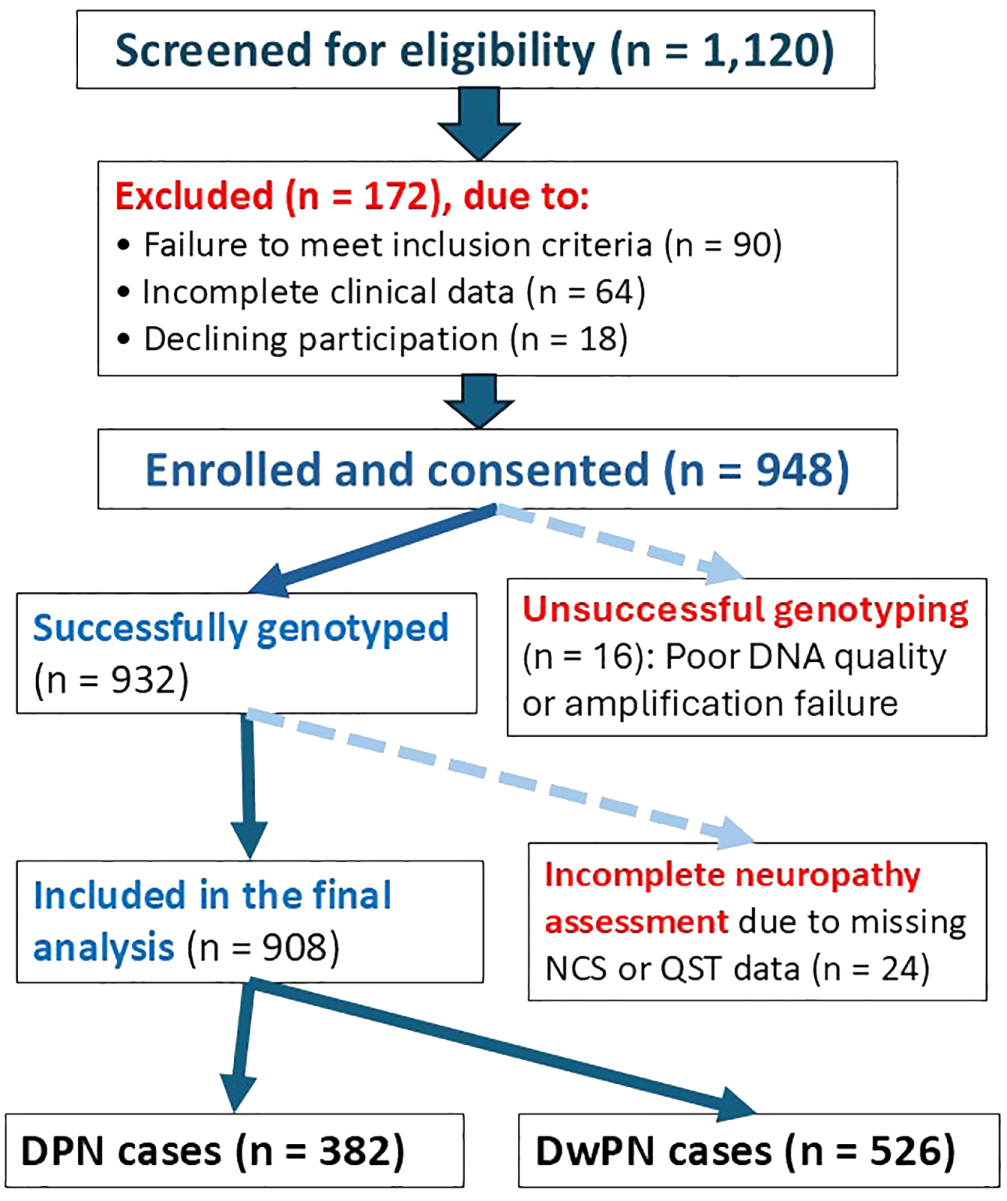
Participant recruitment and analysis flow. Flowchart illustrating the screening, enrollment, genotyping, and final inclusion of participants in the APOE-DPN association study.

Between November 2019 and August 2021, 1,120 unrelated adult Lebanese individuals of Arab descent with type 2 diabetes mellitus (T2DM) were enlisted from outpatient clinics at LAU Medical Center-Rizk Hospital, Rafic Hariri University Hospital, and St. Marc Medical Center in the Greater Beirut area. Of the 1,120 participants screened, 90 were excluded for not meeting inclusion criteria, 64 for incomplete clinical data, and 18 for withdrawing consent (**Figure 2**). Of the 948 enrolled, 16 were excluded due to genotyping failure and 24 for incomplete neuropathy assessment, yielding a final analytical cohort of 908 T2DM patients: 382 with DPN and 526 without (DwPN) (**Figure 2**). Reporting followed STROBE recommendations, with a completed checklist provided in **Supplementary Table S1**.

T2DM was diagnosed based on clinical and laboratory criteria, with no reports of ketoacidosis by the patients. Treatments included diet, oral antidiabetic agents, and insulin; patients who initially required insulin received oral medications for at least 2 years. Blood pressure was checked twice in a seated position, with hypertension defined as BP >140/90 mm Hg on two occasions or the use of antihypertensive medication. Obesity was categorized as a body mass index (BMI) of 30 kg/m² or higher. The control group consisted of 695 healthy, euglycemic Lebanese individuals, matched by gender and geographic origin, with no personal or family history of diabetes. Given the practice of consanguinity in Middle Eastern populations, including Lebanon, we screened for close relatives using family IDs and recruitment records; none were identified, so clustering by family structure was unnecessary. Recorded demographic data included age, gender, ethnicity, BMI, age at diabetes onset, diabetes duration, family history of diabetes, and history of chronic complications and systemic illnesses.

### 2.2 Assessment of diabetic neuropathy

DPN was assessed through clinical, electrophysiological, and patient-reported measures, including Quantitative Sensory Testing (QST) and Nerve Conduction Studies (NCS). Performed by clinicians blinded to genotype status, Nerve Conduction Studies (NCS), and the Michigan Neuropathy Screening Instrument (MNSI), were performed at all study sites. Inter-rater reliability was high (Cohen’s κ = 0.89 for NCS interpretation; κ = 0.85 for clinical examination). Neuropathy assessments and laboratory investigations were conducted during the same clinical visit, with genotyping performed on the same blood sample. All procedures occurred within a 7-day window, ensuring temporal alignment. Clinical evaluation included a structured symptom questionnaire assessing numbness, tingling, and neuropathic pain (burning, shooting, stabbing, or pins-and-needles), accompanied by examination of ankle reflexes, vibration perception (128-Hz tuning fork), and 10-g monofilament sensation. Symptoms were considered present when two or more 2 features were bilateral, distal-predominant, and persistent for more than 3 months. The MNSI questionnaire score was used to quantify symptom severity; scores >4 was deemed abnormal. Ten percent of NCS studies (n = 91) were independently re-reviewed by a second neurologist (κ = 0.89; 95% CI: 0.83–0.95).

QST assessed thermal (cold/warm) thresholds on the dorsum of the foot using the method of limits (1 °C/s ramp; baseline 32 °C), mechanical detection using von Frey filaments, and vibration perception using a biothesiometer. NCS evaluated motor and sensory nerve conduction parameters (amplitude, latency, conduction velocity), with electromyography performed when clinically indicated. The Neuropathy Disability Score (NDS) was computed from clinical and QST findings. Toronto Criterion 2 was fulfilled when two or more motor or sensory nerves demonstrated two or more abnormal electrophysiological parameters. Individuals with alternative causes of neuropathy (vitamin B12 deficiency, hypothyroidism, alcohol abuse, chemotherapy) were excluded.

A standardized multimodal algorithm, aligned with Toronto consensus guidelines, was used to ensure reproducible DPN classification. Diagnosis required abnormal findings in at least two independent modalities: clinical exam, MNSI, QST, or NCS. For NCS, the worst-affected limb was used; studies were considered abnormal if ≥2 parameters in one nerve or ≥1 in two nerves exceeded age-adjusted limits. QST values within ±5% of normative cutoffs were retested in triplicate and deemed abnormal if ≥2 repeated measures fell outside reference ranges. MNSI scores ≥4 were classified as abnormal. Single-modality findings were insufficient. This approach harmonized site-level assessments and reduced misclassification. Pain descriptors and thermal thresholds were available for a subset, allowing limited stratification by pain status and fiber type. No substantial APOE effect differences emerged across these subgroups, though small sample sizes warrant cautious interpretation.

### 2.3 Biochemical analysis

Venous blood samples were drawn via venipuncture from both patients and controls after an overnight fast. Glucose levels were measured using the hexokinase method on Roche Cobas Integra 800 (Mannheim, Germany), while total hemoglobin and HbA1c levels were determined through colorimetric and immunoturbidimetric methods, respectively. Serum lipids, including total cholesterol, HDL, LDL, and triglycerides (TG), were enzymatically measured. Creatinine levels were assessed by the Jaffe reaction, and additional tests of liver and renal function, as well as serum electrolytes, were performed using Dade-Behring instruments.

### 2.4 *APOE* genotyping

Genomic DNA was extracted from the leukocyte-rich layer of EDTA-anticoagulated blood using the phenol-chloroform method. *APOE* genotyping was performed by PCR-RFLP using specific primers and the CfoI restriction enzyme. The digested PCR products were separated on a 5% NuSieve agarose gel. Genotyping quality control measures included running positive control DNAs representing the *APOE* genotypes (ϵ2/ϵ2, ϵ2/ϵ3, ϵ2/ϵ4, ϵ3/ϵ3, ϵ3/ϵ4, ϵ4/ϵ4) in every batch, and re-genotyping a subset of control and case samples by Sanger sequencing; concordance was ≥99%. Call rates exceeded 98% for all samples. Duplicate samples (n = 45, 2.8% of total) demonstrated 100% concordance. The distribution of genotypes in controls satisfied Hardy-Weinberg equilibrium (χ² = 2.14, p = .71). Laboratory personnel were blinded to case-control status during genotyping. Concordance exceeding 99.5% were maintained for inter-plate and intra-plate controls.

### 2.5 Statistical analysis

All analyses were performed using SPSS v29 (IBM, Armonk, NY). Continuous variables are presented as mean ± standard deviation (SD), while categorical variables are shown as counts and percentages. Missing data for covariates (<5%) and *APOE* and DPN (<2%) were minimal. Since the patterns aligned with missing-at-random, we used multiple imputation for variables with >1% missingness, analyzing exposure and outcome as complete cases. Sample size calculations were performed using GPower 3.1.9.7. Based on *APOE* allele frequencies previously reported in Lebanese populations and targeted detectable effect sizes of OR 1.5–2.0 at α = 0.05. pilot data indicating *ϵ4* allele frequencies of 9% in controls and 15% in DPN cases (effect size w = 0.10), we determined that 382 DPN cases and 526 non-DPN controls would provide 85% power to detect this difference at α = 0.05 (two-tailed). Owing to the rarity of *ϵ2/ϵ2* and *ϵ4/ϵ4* (<1%), recessive models were underpowered. Interaction analyses (e.g., ϵ4 × TG) were exploratory and not powered *a priori*, and thus their results should be interpreted with caution. For logistic regression models with 10 covariates and an anticipated OR of 1.5–2.0, our sample size exceeds the recommended minimum of 10 events per variable, providing adequate power (>80%) for multivariable analyses.

Allele and genotype frequencies were calculated by direct gene counting, and the differences between groups were assessed using two-tailed Student’s t-tests (continuous variables) and Pearson’s χ² or Fisher’s exact tests, depending on expected cell counts or sample size (categorical variables), as appropriate. We prespecified a single primary contrast comparing ϵ4 carriers (ϵ3/ϵ4 + ϵ4/ϵ4) with the ϵ3/ϵ3 reference genotype to minimize type I error. Exploratory analyses involving *ϵ2*-containing genotypes and full multi-category models were FDR-corrected, and the Hardy-Weinberg equilibrium (HWE) was assessed separately in controls, DwPN, and DPN groups using exact mid-P correction. *APOE*-DPN analyses were primarily modeled additively, with ϵ2, ϵ3, and ϵ4 dosage assessed via logistic regression. Dominant models grouped ϵ2- (ϵ2/ϵ3, ϵ2/ϵ4) and ϵ4-containing (ϵ3/ϵ4, ϵ4/ϵ4, ϵ2/ϵ4) genotypes against ϵ3/ϵ3 (OR = 1.00). Recessive models (ϵ2/ϵ2, ϵ4/ϵ4) were used only for sensitivity analyses due to low frequencies.

Associations between *APOE* variants and DPN outcomes were examined through multivariate logistic regression, reporting odds ratios (ORs) and 95% confidence intervals (CIs). We compared minimally and lipid-adjusted models, recognizing lipids as mediators and confounders. The primary model included sex, HbA1c, lipids, smoking, hypertension, medications, age at T2DM onset, and duration to assess lipid effects, adjusted for HbA1c/glycemic control. TG were log-transformed due to skewed distributions; values >5.6 mmol/L were flagged, with three retained despite no secondary hypertriglyceridemia. Estimates reflect direct APOE effects, with all models adjusted for statin use. Sensitivity excluding statin users was consistent. Type I error was controlled by grouping analyses: APOE-DPN associations were corrected with Holm-Bonferroni, and lipid/exploratory analyses were corrected with Benjamini-Hochberg FDR. Adjusted p-values are reported, with primary inference based on Holm results. Statistical significance was defined as *p* < 0.05.

We examined effect modification by testing the interaction between ϵ4-carrier status (ϵ3/ϵ4 or ϵ4/ϵ4 vs ϵ3/ϵ3) and log-transformed TG, centered at the sample mean to reduce collinearity, using a logistic regression model adjusted for age, sex, T2DM duration, HbA1c, cholesterol measures, hypertension, smoking, and statin use. Stratum-specific adjusted OR (aORs) were reported for clinically relevant TG categories (<1.7 vs ≥1.7 mmol/L) and validated with TG quartiles. Marginal effects with 95% CIs across the TG distribution were estimated from the interaction model with covariates fixed at sample means or reference levels, employing robust standard errors and a two-sided α=0.05.

### 2.6 Ethics and consent

This study was approved by the Institutional Review Board (IRB) of St. Marc Medical Center (protocol number SMMC-2019-0103, dated October 17, 2019) and adhered to the Declaration of Helsinki. Genetic data were pseudonymized by replacing personal identifiers with unique study codes, with the key securely stored separately and accessible only to authorized personnel. Informed consent was obtained from all participants to ensure confidentiality and compliance with ethical standards. De-identified data underlying the findings, the full data dictionary, analysis code, and workflow documentation are available at the Mendeley Repository: https://doi.org/10.17632/b9mmtpyv4k.1.

## 3 Results

### 3.1 Characteristics of study subjects

**Table 1** summarizes the clinical characteristics of T2DM patients with DPN, those without DPN (DwPN), and normoglycemic controls. While the Lebanese population exhibits low genetic substructure, we tested whether regional variation may bias APOE-DPN associations by stratifying participants by birthplace (North, Mount Lebanon/Beirut, Bekaa/South). While ancestry-informative principal components were unavailable, regional matching and prior population-genetic data suggest that meaningful stratification is unlikely. Between DPN and DwPN, SMDs indicated small imbalances for sex (SMD = 0.16) and HbA1c (SMD = –0.13), and moderate imbalances for age (SMD = 0.40) and TG (SMD = 0.23). Other traits showed minimal differences (|SMD|<0.10). A higher proportion of females (*p* = 0.016) and age (*p* < 0.001) were seen in the DPN group compared to the DwPN and control groups.

**Table 1:** Clinical characteristics of patients and controls.

Characteristic	Controls (n = 695)	DwPN (n = 526)	DPN (n = 382)	*p ^1^*	SMD (DPN vs. DwPN)
Gender (M/F) *^3^*	304 (43.7): 391 (56.3)	260 (49.4): 266 (50.6)	157 (41.2): 225 (58.7) *	0.016	0.163
Age at study (years) *^4^*	57.5 ± 10.8	57.5 ± 11.0	61.8 ± 10.3 *	< 0.001	0.404
Mean BMI (kg/m^2^) *^4^*	23.4 ± 3.3	27.5 ± 4.2 *	27.9 ± 4.4 *	0.118	0.093
Waist-hip ratio *^4^*	0.91 ± 0.08	0.92 ± 0.09	0.94 ± 0.09 *	0.049	0.222
Obesity (>30 kg/m^2^) *^3^*	45 (6.5)	203 (38.2) *	114 (29.6) *	0.005	-0.182
Family history of diabetes *^3^*	N/A	198 (37.2)	132 (34.3)	0.366	0.064
Age of onset (years) *^4^*	N/A	46.2 ± 10.5	47.5 ± 11.3	0.070	0.119
Hypertension *^3^*	108 (15.5)	211 (40.1) *	204 (53.4) *	< 0.001	0.267
SBP (mmHg) *^4^*	121.5 ± 14.1	138.9 ± 28.5 *	143.3 ± 24.6 *	0.014	0.165
DBP (mmHg) *^4^*	78.0 ± 10.5	81.5 ± 12.5 *	82.6 ± 12.7 *	0.199	0.087
Glucose (mmol/L) *^4^*	5.33 ± 0.67	12.89 ± 5.42 *	12.52 ± 5.13 *	0.209	-0.076
HbA1c (%) *^4^*	5.11 ± 1.11	9.61 ± 4.11*	9.12 ± 3.43 *	0.028	-0.133
Total cholesterol (mmol/L) *^4^*	4.89 ± 1.56	5.22 ± 1.54 *	5.31 ± 1.45 *	0.479	0.069
Triglycerides (mmol/L) *^4^*	1.49 ± 0.91	1.71 ± 1.32 *	2.02 ± 1.27 *	0.003	0.231
HDL (mmol/L) *^4^*	1.52 ± 0.89	1.11 ± 0.33 *	1.04 ± 0.31 *	0.508	-0.333
LDL (mmol/L) *^4^*	3.02 ± 1.12	3.78 ± 1.32 *	3.71 ± 1.40 *	0.579	-0.074
Urea (mmol/L) *^4^*	5.40 ± 2.51	7.44 ± 4.03 *	8.78 ± 5.89 *	< 0.001	0.278
Creatinine (μmol/L) *^4^*	61.90 ± 37.7	97.33 ± 67.45 *	101.91 ± 63.02 *	0.294	0.070

DPN, T2DM with neuropathy; DwPN, T2DM without evidence of neuropathy; N/A, not applicable; SMD, Standardized mean differences.

^1^Pearson chi-square (categorical variables); Student *t*-test (continuous variables).

^2^SMD ≥ 0.10 indicates small imbalance and ≥ 0.30 moderate imbalance.

^3^Number of subjects (percent total).

^4^Mean ± SD.

*p < 0.05 *vs.* normoglycemic control.

A higher mean BMI and a higher prevalence of obesity (BMI > 30 kg/m²) were observed in both DPN and DwPN groups compared with controls (*p* = 0.005), but not between T2DM patient subgroups (*p* = 0.118). Hypertension was significantly elevated in both DPN and DwPN groups compared to controls (*p* < 0.001). DPN and DwPN cases were matched for BMI, family history of T2DM, age of onset, fasting glucose, total cholesterol, HDL-cholesterol and LDL-cholesterol. Significant differences between the T2DM subgroups were noted in gender (*p* = 0.016), age at inclusion in the study (*p* < 0.001), waist-hip ratio (*p* = 0.049), hypertension (*p* < 0.001), HbA1c (*p* = 0.028), TG (*p* = 0.003), and urea (*p* < 0.001). While triglyceride levels were significantly higher in the DPN group than in both DwPN and controls (*p* = 0.003), total cholesterol (*p* = 0.479), LDL (*p* = 0.579), and HDL (*p* = 0.508) levels were not significantly different between DPN and DwPN groups compared to controls. In addition, urea levels were significantly elevated in the DPN group compared to DwPN and controls (*p* < 0.001). Creatinine levels were higher in the DPN (101.9 ± 63.0 μmol/L) and DwPN (97.3 ± 67.5 μmol/L) groups than in controls (61.9 ± 37.7 μmol/L). However, this difference was not statistically significant (*p* = 0.294).

### 3.2 Distribution of Apo E alleles and genotypes

The frequencies of the *APOE* alleles among Lebanese were comparable to Middle Eastern populations (Iran, Saudi, Arabia, Egypt and Turkey), as well as South Americans and Africans (**Supplementary Table S2**). Results presented in **Table 2** demonstrated significantly higher *APO-ε2* and *APO-ε4* allele frequencies, but lower APO-*ε*3 allele frequency, among T2DM patients compared to non-diabetic control subjects (all at *p* < 0.001). This resulted from the significantly higher ε2/ε3, ε4/ε4, ε3/ε4, and ε2/ε4, and significantly lower ε3/ε3 genotype frequencies among T2DM cases (all at *p* < 0.001), which assigned positive and negative associations to these genotypes, respectively (**Table 2**). These differences persisted after applying the Bonferroni correction for multiple comparisons, with Holm-adjusted *p*-values reported for primary *APOE*–DPN comparisons and Benjamini-Hochberg FDR-adjusted *p*-values reported for secondary analyses.

**Table 2:** Apolipoprotein E polymorphism in T2DM patients with or without neuropathy.

		Controls (n = 695)	DwPN (n = 526)	DPN (n = 382)	*P ^1^*	OR (95% CI) *^1^*	*Pa* ^2^	OR (95% CI)
Allele	ε2	36 (2.6) ^3^	117 (11.1)	144 (18.8)	**<0.001**	**6.31 (4.42, 9.01**	**<0.001**	**1.86 (1.43, 2.42)**
	ε3	1229 (88.4)	646 (61.4)	392 (51.3)	**<0.001**	**0.17 (0.14, 0.21)**	**<0.001**	**0.66 (0.55, 0.80)**
	ε4	125 (9.0)	289 (27.5)	228 (29.8)	**<0.001**	**4.03 (3.26, 4.97)**	0.269	1.12 (0.91, 1.38)
Genotype	ε3/ε3	561 (80.7)	210 (40.6)	106 (29.1)	**<0.001**	**0.13 (0.10, 0.16)**	**<0.001**	**0.58 (0.44, 0.77)**
	ε2/ε3	16 (2.3)	57 (10.7)	70 (18.4)	**<0.001**	**6.90 (4.06, 11.72)**	**0.001**	**1.85 (1.27, 2.69)**
	ε4/ε4	7 (1.0)	30 (5.3)	22 (5.5)	**<0.001**	**5.97 (2.70, 13.23)**	1.000	1.01 (0.57, 1.78)
	ε3/ε4	91 (13.1)	169 (31.6)	110 (29.1)	**<0.001**	**2.94 (2.27, 3.82)**	0.374	0.88 (0.66, 1.17)
	ε2/ε4	20 (2.9)	60 (11.8)	74 (17.9)	**<0.001**	**5.84 (3.61, 9.45)**	**<0.001**	**1.87 (1.29, 2.70)**

DPN, T2DM with neuropathy; DwPN, T2DM without evidence of neuropathy. Bold face indicates statistically significant differences.

^1^Controls vs. T2DM cases. Adjusted for gender, BMI, hypertension, total cholesterol, TG, HDL, LDL, urea and creatinine.

^2^DPN vs. DwPN. Adjusted for gender, waist-hip ratio, hypertension, HbA1c, TG and urea.

^3^Number (percent total).

The distribution of *APOE* alleles and genotypes was examined in T2DM patients with DPN and those with DwPN, who served as controls. HWE analyses showed that controls (*p* = 0.64) and the DwPN group (p = 0.21) were in equilibrium, and a mild HWE departure was noted for the DPN group (*p* = 0.048). Significantly higher *APO-ε2* (*p* < 0.001) coupled with lower *APO ε3* (*p* < 0.001) allele frequencies were seen in DPN compared to DwPN patient subgroups (**Table 2**). *APO ε4* allele frequency was not significantly different between the two T2DM patient subgroups (*p* = 0.269). Significantly higher frequencies of ε2/ε3 (*p* = 0.001) and ε2/ε4 (*p* < 0.001) and significantly lower frequencies of ε3/ε3 (*p* < 0.001) *APOE* genotypes were seen among DPN cases, which conferred DPN-susceptible and -protective nature to these genotypes, respectively (**Table 2**). Compared to the *ϵ3* allele, both *ϵ2* and *ϵ4* alleles conferred increased odds of DPN.

**Figure 3:**
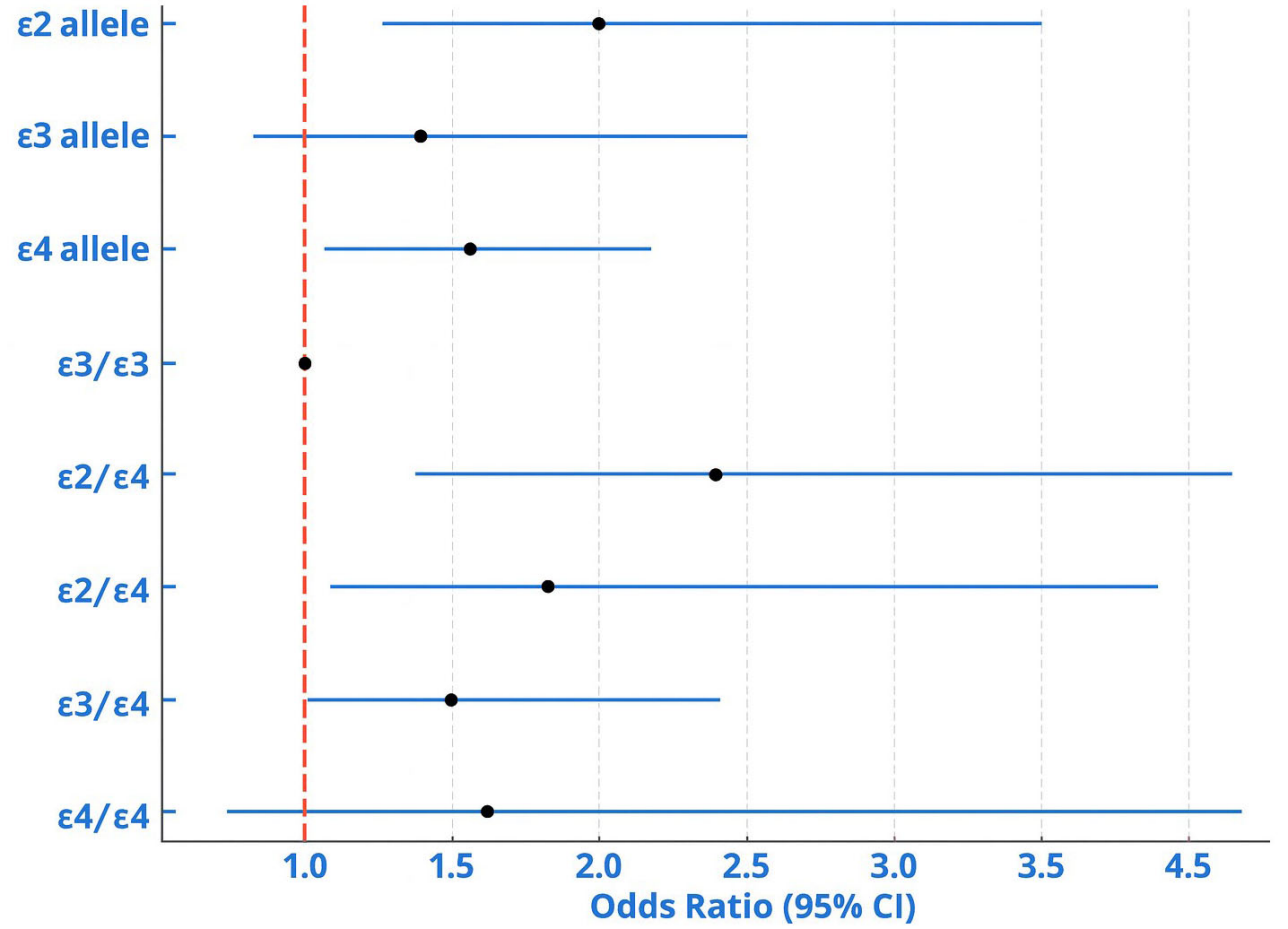
Forest plot showing ORs with 95% CIs for *ApoE* alleles and genotypes associated with DPN. Both *ϵ2* and *ϵ4* alleles, as well as *ϵ2*/*ϵ3*, *ϵ2*/*ϵ4*, and *ϵ3*/*ϵ4* genotypes, were significantly linked to increased DPN risk, while *ϵ4*/*ϵ4* showed a nonsignificant trend.

At the genotype level, *ϵ2/ϵ3* (OR = 1.85 [1.27–2.69]), ϵ2/ϵ4 (OR = 1.87 [1.29–2.70]), and *ϵ3/ϵ4* (OR = 1.62 [1.08–2.44]) were significantly associated with increased odds of DPN, while *ϵ4/*ϵ4 (OR = 1.01 [0.57–1.78]) showed no significant effect (**Figure 3**). In logistic regression, ϵ4 carriers (ϵ3/ϵ4+ϵ4/ϵ4) had higher odds of DPN versus ϵ3/ϵ3 in the minimally adjusted model (age, sex, duration, HbA1c) and the lipid-adjusted model (adding total cholesterol, HDL, LDL, log-TG, hypertension, smoking, statin use) (report aOR, 95% CI, p for both models). Pooled ϵ2-containing genotypes (ϵ2/ϵ3+ϵ2/ϵ4) also showed higher odds (**Figure 3**).

### 3.3 Effect on lipid profile

Genotype-specific lipid patterns are shown in **Table 3**. Compared to DwPN cases, DPN cases carrying the ϵ2/ϵ3 genotype had significantly higher total cholesterol (p = .018) and LDL-cholesterol (*p* = 0.045), and lower HDL-cholesterol (*p* = 0.042) (**Table 3**). Similarly, ϵ3/ϵ4 genotype was linked to significantly higher total cholesterol (*p* = 0.002) and TG (*p* < 0.001) (**Table 3**). DPN cases with ϵ3-carrying genotypes (ϵ3/ϵ3 + ϵ2/ϵ3 + ϵ3/ϵ4) had higher total cholesterol (*p* = 0.030) and TG (*p* < 0.001), but lower HDL-cholesterol (*p* = 0.017), while ϵ4-carrying genotypes (ϵ4/ϵ4 + ϵ3/ϵ4 + ϵ2/ϵ4) showed similar trends (*p* = 0.004 for cholesterol; *p* < 0.001 for TG and *p* = 0.032 for HDL-cholesterol). Additionally, higher LDL-cholesterol was associated with ϵ3-carrying genotypes in the Apo E3 group (*p* = 0.009) and the ϵ2/ϵ3 genotype (*p* = 0.045). A significant ϵ4 × log(TG) interaction was observed, with ϵ4 carriers showing higher DPN odds in the high TG group (≥1.7 mmol/L) but attenuated association in the low/normal group (<1.7 mmol/L); marginal effects indicated a monotonic rise in ϵ4-related risk across TG, reaching significance near the upper tertile. Sensitivity analyses confirmed robustness, with exclusion of lipid-lowering therapy users or lipid adjustment yielding similar or stronger effects, suggesting partial lipid mediation. Results were unchanged with stricter DPN definitions or exclusion of extreme TG values (>5.6 mmol/L), and sex-stratified models showed comparable effects in men and women.

**Table 3:** Serum lipid profiles by apolipoprotein E genotypes.

Alleles and Genotypes	Total cholesterol ^1^			Triglycerides ^1^			HDL-cholesterol ^1^			LDL-cholesterol ^1^		
	DwPN	DPN	*P*	DwPN	DPN	*P*	DwPN	DPN	*P*	DwPN	DPN	*P*
E2	5.21 ± 1.20	5.23 ± 1.41	0.990	1.65 ± 1.02	2.01 ± 1.34	0.072	1.17 ± 0.44	1.11 ± 0.43	0.360	3.33 ± 1.30	3.3 ± 1.33	0.744
E3	5.32 ± 1.42	5.44 ± 1.54	0.112	**2.04 ± 1.41**	**1.70 ± 1.32**	**0.002**	1.00 ± 0.31	1.01 ± 0.32	0.882	4.02 ± 1.32	4.01 ± 1.30	0.811
E4	5.34 ± 1.30	5.42 ± 1.62	0.310	**2.11 ± 1.42**	**1.71 ± 1.31**	**0.003**	1.11 ± 0.32	1.02 ± 0.33	0.220	3.72 ± 1.51	3.84 ± 1.53	0.760
ε3/ε3	5.24 ± 1.44	5.14 ± 1.41	0.454	1.82 ± 1.41	1.83 ± 1.23	0.840	1.02 ± 0.31	1.00 ± 0.20	0.671	4.22 ± 1.12	4.51 ± 0.93	0.353
ε2/ε3	5.44 ± 1.21	5.50 ± 1.42	0.70	1.81 ± 1.12	2.11 ± 1.33	0.140	1.11 ± 0.33	1.10 ± 0.44	0.971	3.22 ± 1.22	3.41 ± 1.23	0.611
ε4/ε4	5.22 ± 1.10	4.61 ± 1.33	0.091	1.89 ± 1.31	1.78 ± 1.11	0.729	1.09 ± 0.44	1.10 ± 0.27	0.758	2.89 ± 1.21	2.43 ± 1.24	0.310
ε3/ε4	**5.28 ± 1.36**	**5.78 ± 1.62**	**0.010**	**1.67 ± 1.40**	**2.33 ± 1.45**	**0.001**	1.00 ± 0.21	1.01 ± 0.28	0.592	4.04 ± 1.56	4.31 ± 1.44	0.322
ε2/ε4	5.08 ± 1.19	5.02 ± 1.37	0.610	1.56 ± 1.02	1.78 ± 1.20	0.321	1.18 ± 0.43	1.11 ± 0.42	0.187	3.45 ± 1.30	3.01 ± 1.30	0.267

DPN, T2DM with neuropathy; DwPN, T2DM without evidence of neuropathy. Bold face indicates statistically significant differences.

4. Mean ± SD of lipids (mmol/L).

5. E2 = ϵ2/ϵ3 + ϵ2/ϵ4; E3 = ϵ3/ϵ3 + ϵ2/ϵ3 + ϵ3/ϵ4; E4 = ϵ4/ϵ4 + ϵ3/ϵ4 + ϵ2/ϵ4.

### 3.4 Association between APOE genotypes and increased odds of DPN

Logistic regression was performed with two adjustment models. Model 1 adjusted for age of onset, lipid profile, fasting glucose, and HbA1c, while Model 2 additionally adjusted for nephropathy, retinopathy, gender, and hypertension (**Figure 3**). *Apo ϵ2*-containing genotype group (*ϵ2*/*ϵ3* + *ϵ2*/*ϵ4*) were not significantly associated with altered odds of DPN according to Model 1 (*p* = 0.343) and Model 2 (*p* = 0.196). Although not statistically significant, the Apo ϵ3-containing genotype group (ϵ3/ϵ3+ϵ2/ϵ3+ϵ3/ϵ4) indicated a trend towards increased odds of DPN in Model 1 (*p* = 0.092) and Model 2 (*p* = 0.065). In contrast, the Apo ϵ4-containing genotype group (ϵ4/ϵ4+ϵ3/ϵ4+ϵ2/ϵ4) was significantly associated with a higher odds of DPN in both Model 1 (*p* = 0.013) and Model 2 (*p* = 0.028).

### 3.5 Correlation between *APOE* genotypes and DPN and associated features

**Figure 1** illustrates the correlations between *APOE* genotypes and clinical features of DPN. Significant positive correlations were observed between ϵ2/ϵ3 and both retinopathy (ρ = 0.60, *p* < 0.01) and diabetes duration (ρ = 0.54, *p* < 0.05). The ϵ4/ϵ4 genotype showed a strong positive correlation with BMI (ρ = 0.68, p <.05) but an inverse association with disease duration (ρ = –0.72, *p* < 0.05). Conversely, the ϵ3/ϵ3 and ϵ2/ϵ4 genotypes demonstrated weaker or nonsignificant correlations across most clinical parameters. These results highlight distinct genotype-specific patterns that influence DPN-related clinical characteristics.

Stratified analyses and formal interaction testing were performed to evaluate the contribution of key clinical factors to the *APOE* effects on DPN risk. Consistent association between *ϵ4*-containing genotypes and increased odds of DPN was noted across the diabetes low duration (<10 years: OR [95% CI] = 1.58 [1.12–2.23]) and prolonged duration (≥10 years: OR [95% CI] = 1.67 [1.21–2.31]) strata (*p* interaction = 0.76), HbA1c categories (<8%: OR = 1.61; ≥8%: OR = 1.64; *p* interaction = 0.89), and hypertension (present: OR = 1.59; absent: OR = 1.66; *p* interaction = 0.82). A significant interaction was observed with hypertriglyceridemia status (TG >2.0 mmol/L), in which ϵ4 effects were amplified among hypertriglyceridemic patients (OR = 2.34, 95% CI: 1.62–3.38) compared to normotriglyceridemic patients (OR [95% CI] = 1.21 [0.78–1.87], *p* interaction = 0.03), suggesting that lipid-mediated mechanisms may partially explain the association between ϵ4 variants and altered odds of DPN. Notably, these associations persisted after adjustment for HbA1c, suggesting that lipid pathways may operate independently of glycemic control to modulate genetic susceptibility to DPN.

## 4 Discussion

This study examined the relationship between *APOE* polymorphisms and the increased odds of DPN in Lebanese patients with T2DM. It is the first large-scale study in the Middle East to link APOE polymorphisms to DPN, thereby broadening global understanding of ancestry-specific genetic risk. While the modest HWE deviation observed in the DPN subgroup may suggest genotyping or sampling imprecision, this is unlikely given that the DwPN cases were in HWE, prompting speculation about a disease association. Our results show a strong association between certain *APOE* genotypes and DPN susceptibility, with ϵ4-containing genotypes showing the strongest association with higher odds of DPN, after adjustment for traditional metabolic and clinical factors. Consistent effect estimates across sensitivity checks support the stability of the *APOE*-DPN association. These findings are particularly important within the Lebanese population, where extensive genetic admixture and diversity may affect the expression and impact of *APOE* variants (36), offering new insights into ethnicity-specific risk factors (37, 38). The results both support and differ from findings of studies investigating *APOE* polymorphisms in T2DM across various populations, underlining the importance of exploring genetic contributions to DPN within specific ethnic groups to better understand shared mechanisms and population-specific vulnerabilities (39).

Extended mechanistic pathways linking *APOE* isoforms to oxidative stress, microvascular dysfunction, and neuroinflammatory injury are provided in **Supplementary Note 1**. Our observation of higher ϵ2 and ϵ4 allele frequencies in Lebanese T2DM patients compared to normoglycemic controls aligns with previous reports linking these variants to metabolic dysregulation, dyslipidemia, and cardiovascular risk (26, 40), despite considerable variation in effect sizes and population-specific patterns. Conversely, the *ϵ3* allele, considered metabolically neutral (1, 21), was underrepresented in our DPN patients, consistent with earlier studies in Caucasian and Asian cohorts (41–43) and reinforcing its potential protective role. This was reminiscent of earlier Chinese studies, which documented significant associations of the ϵ4 allele with increased odds of DPN, while the ϵ3 allele was protective (44). A more recent Chinese study confirmed the significant association between DPN and ϵ4, although with effect sizes that were more modest than in our Lebanese population or the cohort of Tang et al. (45).

An earlier Japanese study involving 158 patients with T2DM reported increased frequency and severity of neuropathy in ϵ4 carriers compared with ϵ3 or ϵ2 carriers, despite similar age, BMI, HbA1c, or diabetes duration (46). A US-based study of 187 patients with diabetes corroborated this link. It demonstrated that *ϵ4*-containing genotypes (*ϵ3*/*ϵ4*, *ϵ4*/*ϵ4*) show differing associations with severe neuropathy on the NIS-LL (Neuropathy Impairment Score in the Lower Limbs), independent of glycemia or TG, compared with other genotypes (47). Furthermore, a Greek study reported an association between ϵ4 carriage and a 5-fold increased risk of severe neuropathy (48). Noteworthy was the association with *ϵ4*/*ϵ4* + *ϵ3*/*ϵ4* genotype ocular impairments caused by NAION (nonarteritic anterior ischemic optic neuropathy) (49).

Although our findings support an association between *APOE ϵ4* and DPN, several studies have reported null or discordant results. While a Chinese study identified ϵ3 or ϵ2, rather than ϵ4, as the primary variant of interest (50), Zhou et al. found no *ϵ4* differences between diabetic neuropathy cases and controls (51). Similarly, studies on Swedish and Russian cohorts reported absent or weak associations (52, 53). These seemingly conflicting associations of *APOE* genotypes with altered odds of neuropathy may be due to population-specific modifier genes, baseline metabolic profiles, population-specific LD patterns and consanguinity effects in Middle Eastern vs. Northern European or East Asian cohorts (36, 37), environmental factors, diabetes type-specific pathophysiology (T1DM vs T2DM), and variations in neuropathy assessment criteria (1, 35). This highlights the need for standardized protocols and larger, multi-ethnic cohorts to clearly determine the role of *APOE* in diabetes and related complications.

The differential lipid profiles associated with *APOE* genotypes in our DPN patients provide mechanistic insights into DPN pathogenesis (16, 54). Interpretation focuses on the pre-specified *ϵ4* carrier versus ϵ3/ϵ3 contrast; all other genotype comparisons were exploratory and should be viewed cautiously given FDR-controlled multiple testing. The significant elevation of total cholesterol and LDL-cholesterol levels in ϵ2/ϵ3 genotype-carrying DPN cases suggests lipotoxicity linked to neural tissue (6), while atherogenesis in ϵ4-containing genotypes, highlighted by elevated cholesterol and TG, and reduced HDL-cholesterol, is consistent with the microvascular dysfunction hypotheses (7, 9). The association between ϵ4 genotypes and dyslipidemia is consistent with earlier findings that link this allele to impaired lipid clearance (55, 56). Noteworthy was the association of the ϵ2/ϵ3 genotype with unfavorable lipid profiles, which challenges the conventional view of ϵ2’s protective effects (23). This suggests that diabetic states may modulate APOE metabolism (57). These findings underscore the importance of genetic predisposition in assessing cardiovascular and microvascular risk in DPN, suggesting that the underlying disease state may dictate the effects of different *APOE* alleles on lipid metabolism.

**Figure 4:**
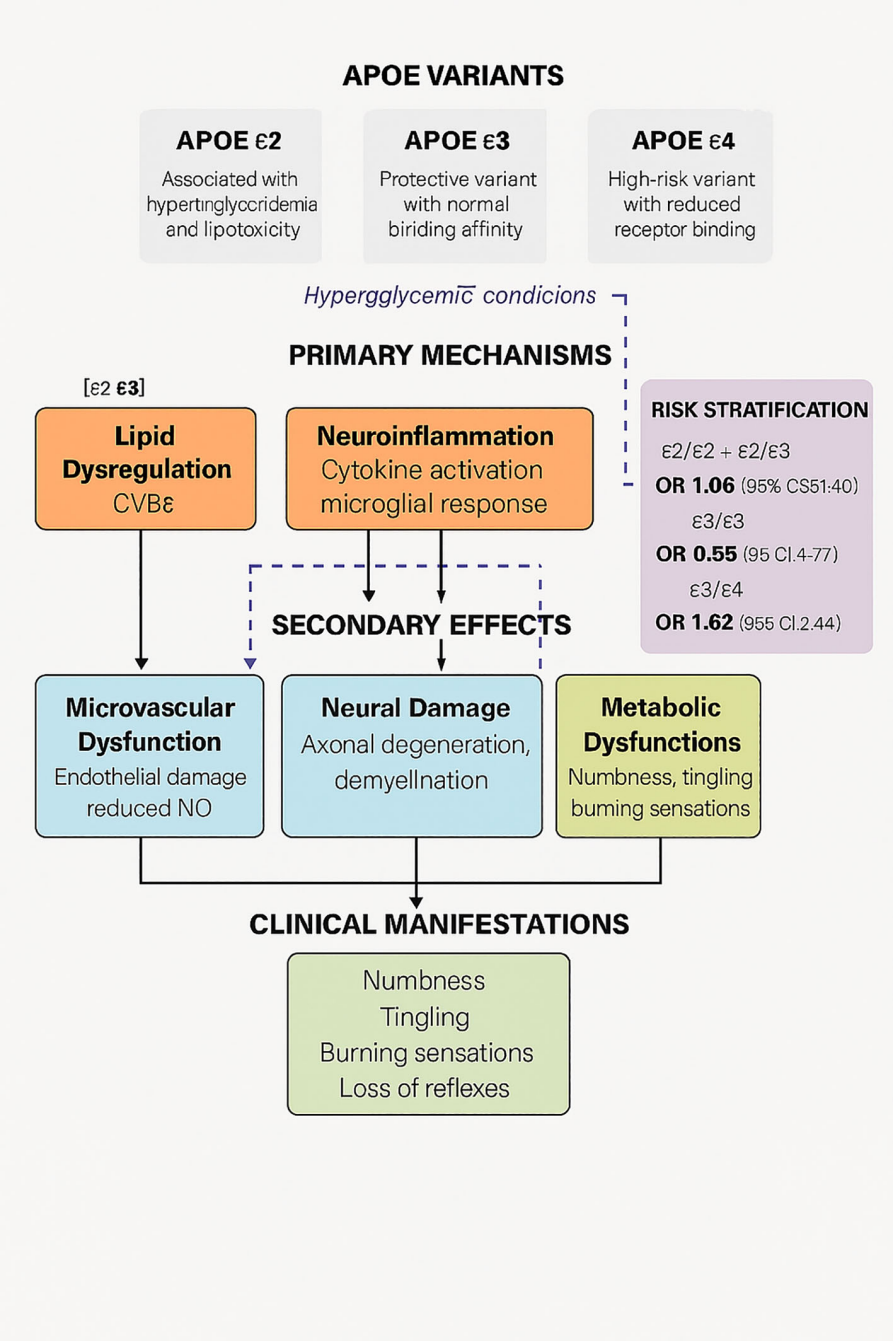
Hypothesized pathways linking *APOE* polymorphisms to DPN development. This conceptual model illustrates proposed mechanisms through which APOE ϵ2, ϵ3, ϵ4 variants may influence DPN risk under hyperglycemic conditions. Lipid dysregulation, neuroinflammation, and oxidative stress are shown to contribute to secondary effects including microvascular dysfunction, neural damage, and metabolic disturbances. Arrows are schematic and do not imply causality established by this study. AGE, advanced glycation end products; NO, nitric oxide; ROS, reactive oxygen species. [Based on Mahley (18), Liu et al. (50), and Tudorache (20)].

Although APOE isoforms plausibly influence DPN through lipid dysregulation, oxidative stress, and microvascular injury (**Figure 4**), our cross-sectional design cannot establish whether genotype-associated lipid alterations precede or follow neuropathy onset; the noted associations suggest potential mechanistic pathways. In this study, we emphasize interpretation rather than mechanistic restatement: the more substantial effect of ϵ4-containing genotypes, together with the ϵ4-triglyceride interaction, suggests that lipid-mediated microvascular and neuroinflammatory pathways may amplify neurodegeneration in susceptible individuals (25, 44, 48, 58). Full mechanistic context and pathway-level explanations are now provided in **Supplementary Note 1** and **Figure 4**.

Despite its association with other diabetic complications, such as retinopathy, the protective effect of ϵ3/ϵ3 genotype in DPN suggests tissue-specific mechanisms of *APOE* action (33, 43, 44) (**Supplementary Figure 1**). The ϵ3/ϵ3 genotype, typically considered metabolically neutral (21), correlated positively with retinopathy and hypertension but negatively with nephropathy. Notably, the enrichment of ϵ2/ϵ3 and ϵ2/ϵ4 among DPN patients, without consistent associations with other complications, suggests that ϵ2 may act through neuropathy-specific mechanisms involving impaired neuronal repair or inflammatory modulation (27). Clinically, *APOE* typing may identify high-risk patients who could benefit from intensified lipid control and neuroprotective interventions (1, 59), aligning with the precision-medicine agenda in diabetes care. Our findings show a statistical association between *APOE*-related lipid metabolism and DPN, adjusting for HbA1c/glycemic control, reinforcing the role of lipid pathways in neuropathic risk (60). Future studies should clarify these relationships through prospective designs, multi-ethnic replication, mediation analyses, and gene-drug interaction testing.

This study has several strengths. It is one of the most comprehensive investigations of *APOE* and DPN in a Middle Eastern cohort, involving patients who underwent detailed phenotyping using multimodal neuropathy assessment (QST, NCS, MNSI), and analyses employing rigorous multivariable logistic regression that adjusts for multiple confounders. The inclusion of the ethnically homogeneous Lebanese population further improved reliability and provided new insights into an underrepresented group in global diabetes genetics studies. However, there are also limitations to consider when interpreting our results. The cross-sectional design precludes establishing temporality, and thus all *APOE*-DPN associations should be interpreted as correlational rather than causal. While the timing of lipid changes relative to nerve damage also remains unclear (61, 62), we recognize that lipid levels may partially mediate the direct *APOE* effects while also acting as confounded traits influenced by treatment (statins, fibrates, neuroactive agents) and metabolic factors. Accordingly, lipid-adjusted models reflect mediation-controlled, not total, effects (63).

In addition, hospital-based recruitment may introduce selection bias and overestimate effect sizes, and the single-population design limits generalizability. Clinic samples also risk severity and survivorship bias, as tertiary-care patients typically have more advanced or treatment-engaged neuropathy, while mild, early, or undiagnosed cases, along with patients lost to follow-up, are underrepresented. These factors may shift genotype-phenotype patterns and underscore the need for community-based, multi-ethnic studies. Furthermore, while we acknowledge the absence of ancestry-marker PCA, region-stratified analyses, and the known homogeneity of the Lebanese population, these suggest minimal confounding from population substructure. Furthermore, reliance on a single HbA1c measure limits the assessment of long-term, variable glycemic exposure, prompting speculation about residual confounding. We also lacked statistical power to assess gene-medication (e.g., statin treatment) interactions and had limited data on pain status and thermal thresholds, which prevented adequately powered analyses of DPN subtypes, thus preventing assessment of the potential heterogeneity in APOE associations.

Statin use was accounted for, and sensitivity analyses confirmed stable *APOE*-DPN associations, but missing data on other cardiometabolic drugs and lifestyle or socioeconomic factors, such as diet, activity, alcohol, smoking intensity (types and numbers/day) and duration (including former and passive smokers), and education, may have introduced residual confounding through their impact on lipid levels and microvascular risk. Lastly, neuropathy-specific medications (duloxetine, pregabalin, and others) were not consistently recorded across sites; their omission may contribute to residual confounding, as their use may correlate with symptom severity and management. Nonetheless, these limitations do not lessen the significance of the strong associations observed, which remain significant after rigorous adjustment. Longitudinal follow-up and functional validation of genetic variants will further refine mechanistic understanding and APOE’s potential for identifying high-risk individuals.

## 5 Conclusion

In summary, our cross-sectional study demonstrates that *APOE* genetic polymorphisms, particularly ϵ4-containing genotypes, are significantly associated with higher odds of DPN in this hospital-based T2DM cohort, particularly in relation to lipid abnormalities. These associations persist after adjustment for traditional risk factors, including glycemic control, establishing *APOE* variants as essential markers of DPN susceptibility. While the results raise the possibility that *APOE*-linked metabolic pathways contribute to neuropathic risk, they remain exploratory and require independent confirmation, given their cross-sectional design, their focus on a single population (Lebanese), and the inability to establish temporal relationships between genotype, dyslipidemia, and neuropathy. Finally, it should be noted that the observed independence from glycemic control reflects statistical adjustment, not true causality, as unmeasured confounding and the cross-sectional design limit causal inference. Prospective longitudinal studies in diverse populations are needed to determine causality, establish whether lipid-mediated pathways represent modifiable therapeutic targets, and explore potential therapeutic implications.

## Data Availability

The datasets presented in this study can be found online at the Mendeley repository: https://doi.org/10.17632/b9mmtpyv4k.1.

## Supplementary material

Supplementary Figure 1 Directed acyclic graph (DAG) illustrating the hypothesized causal structure for the association between APOE genotype and diabetic peripheral neuropathy (DPN).

## Glossary

ACEi: Angiotensin-converting enzyme inhibitor
ARB: Angiotensin II receptor blocker
APOE: Apolipoprotein E
BMI: Body mass index
CI: Confidence interval
DBP: Diastolic blood pressure
DAG: Directed acyclic graph
DPN: Diabetic peripheral neuropathy
DwPN: Diabetes without peripheral neuropathy
EMG: Electromyography
FDR: False discovery rate
HDL: High-density lipoprotein
LDL: Low-density lipoprotein
MNSI: Michigan Neuropathy Screening Instrument
NCS: Nerve conduction studies
NDS: Neuropathy Disability Score
OR: Odds ratio
QST: Quantitative sensory testing
SMD: Standardized mean difference
SBP: Systolic blood pressure
SNP: Single nucleotide polymorphism
TG: Triglycerides

## References

[1] Atageldiyeva KK Nemr R Echtay A Racoubian E Sarray S Almawi WY . Apolipoprotein E genetic polymorphism influence the susceptibility to nephropathy in type 2 diabetes patients. Gene. (2019) 715:144011. doi: 10.1016/j.gene.2019.144011, PMID: 31357022

[2] Galicia-Garcia U Benito-Vicente A Jebari S Larrea-Sebal A Siddiqi H Uribe KB . Pathophysiology of type 2 diabetes mellitus. Int J Mol Sci. (2020) 21:6275. doi: 10.3390/ijms21176275, PMID: 32872570 PMC7503727

[3] International Diabetes Federation . IDF Diabetes Atlas. 10th edition. Brussels: International Diabetes Federation (2021).

[4] Hossain MJ Al-Mamun M Islam MR . Diabetes mellitus, the fastest growing global public health concern: Early detection should be focused. Health Sci Rep. (2024) 7:e2004. doi: 10.1002/hsr2.2004, PMID: 38524769 PMC10958528

[5] Sellami N Lamine LB Turki A Sarray S Jailani M Al-Ansari AK . Association of VEGFA variants with altered VEGF secretion and type 2 diabetes: A case-control study. Cytokine. (2018) 106:29–34. doi: 10.1016/j.cyto.2018.03.003, PMID: 29533820

[6] Feldman EL Callaghan BC Pop-Busui R Zochodne DW Wright DE Bennett DL . Diabetic neuropathy. Nat Rev Dis Primers. (2019) 5:42. doi: 10.1038/s41572-019-0097-9, PMID: 31197183 PMC7096070

[7] Pop-Busui R Boulton AJ Feldman EL Bril V Freeman R Malik RA . Diabetic neuropathy: A position statement by the American Diabetes Association. Diabetes Care. (2017) 40:136–54. doi: 10.2337/dc16-2042, PMID: 27999003 PMC6977405

[8] Galiero R Caturano A Vetrano E Beccia D Brin C Alfano M . Peripheral neuropathy in diabetes mellitus: Pathogenetic mechanisms and diagnostic options. Int J Mol Sci. (2023) 24:3554. doi: 10.3390/ijms24043554, PMID: 36834971 PMC9967934

[9] Zhu J Hu Z Luo Y Liu Y Luo W Du X . Diabetic peripheral neuropathy: pathogenetic mechanisms and treatment. Front Endocrinol (Lausanne). (2024) 14:1265372. doi: 10.3389/fendo.2023.1265372, PMID: 38264279 PMC10803883

[10] Preston FG Riley DR Azmi S Alam U . Painful diabetic peripheral neuropathy: Practical guidance and challenges for clinical management. Diabetes Metab Syndr Obes. (2023) 16:1595–612. doi: 10.2147/DMSO.S370050, PMID: 37288250 PMC10243347

[11] Zhou P Zhou JS Li JJ Qin L Hu WF Zhang XY . Prevalence and risk factors for painful diabetic peripheral neuropathy: a systematic review and meta-analysis. Front Neurol. (2025) 16:1564867. doi: 10.3389/fneur.2025.1564867, PMID: 40433609 PMC12108811

[12] Lee JE Won JC . Clinical phenotypes of diabetic peripheral neuropathy: Implications for phenotypic-based therapeutics strategies. Diabetes Metab J. (2025) 49:542–64. doi: 10.4093/dmj.2025.0299, PMID: 41243306 PMC12620676

[13] Ang L Jaiswal M Martin C Pop-Busui R . Glucose control and diabetic neuropathy: lessons from recent large clinical trials. Curr Diabetes Rep. (2014) 14:528. doi: 10.1007/s11892-014-0528-7, PMID: 25139473 PMC5084623

[14] Al Yafei Z Mack SJ Alvares M Ali BR Afandi B Beshyah SA . HLA-DRB1 and -DQB1 alleles, haplotypes and genotypes in Emirati patients with type 1 diabetes underscores the benefits of evaluating understudied populations. Front Genet. (2022) 13:841879. doi: 10.3389/fgene.2022.841879, PMID: 35419034 PMC8997289

[15] Nemr R Salman RA Jawad LH Juma EA Keleshian SH Almawi WY . Differential contribution of MTHFR C677T variant to the risk of diabetic nephropathy in Lebanese and Bahraini Arabs. Clin Chem Lab Med. (2010) 48:1091–4. doi: 10.1515/CCLM.2010.228, PMID: 20524928

[16] Martínez-Martínez AB Torres-Perez E Devanney N Del Moral R Johnson LA Arbones-Mainar JM . Beyond the CNS: The many peripheral roles of APOE. Neurobiol Dis. (2020) 138:104809. doi: 10.1016/j.nbd.2020.104809, PMID: 32087284 PMC7195217

[17] Vinelli-Arzubiaga D Suasnabar Campos CE Laso-Salazar MC Abarca-Barriga H . Polymorphic variants and risk of diabetic peripheral neuropathy in patients with type 2 diabetes mellitus: systematic review and meta-analysis. BMC Endocr Disord. (2025) 25:69. doi: 10.1186/s12902-025-01897-1, PMID: 40082898 PMC11907806

[18] Mahley RW . Apolipoprotein E: Cholesterol transport protein with expanding role in cell biology. Sci Sci. (1988) 240:622–30. doi: 10.1126/science.3283935, PMID: 3283935

[19] Belloy ME Napolioni V Greicius MD . A quarter century of APOE and Alzheimer’s Disease: Progress to date and the path forward. Neuron. (2019) 101:820–38. doi: 10.1016/j.neuron.2019.01.056, PMID: 30844401 PMC6407643

[20] Tudorache IF Trusca VG Gafencu AV . Apolipoprotein E - A multifunctional protein with implications in various pathologies as a result of its structural features. Comput Struct Biotechnol J. (2017) 15:359–65. doi: 10.1016/j.csbj.2017.05.003, PMID: 28660014 PMC5476973

[21] Lumsden AL Mulugeta A Zhou A Hyppönen E . Apolipoprotein E (APOE) genotype-associated disease risks: a phenome-wide, registry-based, case-control study utilising the UK Biobank. EBioMedicine. (2020) 59:102954. doi: 10.1016/j.ebiom.2020.102954, PMID: 32818802 PMC7452404

[22] Saidi S Slamia LB Ammou SB Mahjoub T Almawi WY . Association of apolipoprotein E gene polymorphism with ischemic stroke involving large-vessel disease and its relation to serum lipid levels. J Stroke Cerebrovasc Dis. (2007) 16:160–6. doi: 10.1016/j.jstrokecerebrovasdis.2007.03.001, PMID: 17689412

[23] El-Lebedy D Raslan HM Mohammed AM . Apolipoprotein E gene polymorphism and risk of type 2 diabetes and cardiovascular disease. Cardiovasc Diabetol. (2016) 15:12. doi: 10.1186/s12933-016-0329-1, PMID: 26800892 PMC4724147

[24] Phillips MC . Is ABCA1 a lipid transfer protein? J Lipid Res. (2018) 59:749–63. doi: 10.1194/jlr.R082313, PMID: 29305383 PMC5928442

[25] Yang LG March ZM Stephenson RA Narayan PS . Apolipoprotein E in lipid metabolism and neurodegenerative disease. Trends Endocrinol Metab. (2023) 34:430–45. doi: 10.1016/j.tem.2023.05.002, PMID: 37357100 PMC10365028

[26] Dias D Portugal CC Relvas J Socodato R . From genetics to neuroinflammation: the impact of ApoE4 on microglial function in Alzheimer’s Disease. Cells. (2025) 14:243. doi: 10.3390/cells14040243, PMID: 39996715 PMC11853365

[27] Li Z Shue F Zhao N Shinohara M Bu G . APOE2: protective mechanism and therapeutic implications for Alzheimer’s disease. Mol Neurodegener. (2020) 15:63. doi: 10.1186/s13024-020-00413-4, PMID: 33148290 PMC7640652

[28] Shvetcov A Johnson ECB Winchester LM Walker KA Wilkins HM Thompson TG . APOE ϵ4 carriers share immune-related proteomic changes across neurodegenerative diseases. Nat Med. (2025) 31:2590–601. doi: 10.1038/s41591-025-03835-z, PMID: 40665049 PMC12353839

[29] Vaisi-Raygani A Rahimi Z Nomani H Tavilani H Pourmotabbed T . The presence of apolipoprotein ϵ4 and ϵ2 alleles augments the risk of coronary artery disease in type 2 diabetic patients. Clin Biochem. (2007) 40:1150–6. doi: 10.1016/j.clinbiochem.2007.06.010, PMID: 17689519

[30] Mahley RW . Central nervous system lipid abnormalities in APOE knockout mice. Neurobiol Dis. (2016) 96:30–9.

[31] Sheng L Yang Y Zhou Y . Association between lipoprotein(a) and diabetic peripheral neuropathy in patients with type 2 diabetes: a meta-analysis. Diabetol Metab Syndr. (2025) 17:76. doi: 10.1186/s13098-025-01621-y, PMID: 40033299 PMC11877928

[32] Al-Jenaidi FA Wakim-Ghorayeb SF Al-Abbasi A Arekat MR Irani-Hakime N Najm P . Contribution of selective HLA-DRB1/DQB1 alleles and haplotypes to the genetic susceptibility of type 1 diabetes among Lebanese and Bahraini Arabs. J Clin Endocrinol Metab. (2005) 90:5104–9. doi: 10.1210/jc.2005-1166, PMID: 15985473

[33] Chen W Li B Wang H Wei G Chen K Wang W . Apolipoprotein E E3/E4 genotype is associated with an increased risk of type 2 diabetes mellitus complicated with coronary artery disease. BMC Cardiovasc Disord. (2024) 24:160. doi: 10.1186/s12872-024-03831-0, PMID: 38491412 PMC10941446

[34] Tai LM Thomas R Marottoli FM Koster KP Kanekiyo T Morris AW . The role of APOE in cerebrovascular dysfunction. Acta Neuropathol. (2016) 131:709–23. doi: 10.1007/s00401-016-1547-z, PMID: 26884068 PMC4837016

[35] Almawi WY Nemr R Keleshian SH Echtay A Saldanha FL AlDoseri FA . A replication study of 19 GWAS-validated type 2 diabetes at-risk variants in the Lebanese population. Diabetes Res Clin Pract. (2013) 102:117–22. doi: 10.1016/j.diabres.2013.09.001, PMID: 24145053

[36] El Shamieh S Costanian C Kassir R Visvkis-Siest S Bissar-Tadmouri N . APOE genotypes in Lebanon: distribution and association with hypercholesterolemia and Alzheimer’s disease. Per Med. (2019) 16:15–23. doi: 10.2217/pme-2018-0067, PMID: 30457419

[37] Almawi WY Nemr R Finan RR Saldhana FL Hajjej A . HLA-A, -B, -C, -DRB1 and -DQB1 allele and haplotype frequencies in Lebanese and their relatedness to neighboring and distant populations. BMC Genomics. (2022) 23:456. doi: 10.1186/s12864-022-08682-7, PMID: 35725365 PMC9208108

[38] Jalkh N Mehawej C Chouery E . Actionable exomic secondary findings in 280 lebanese participants. Front Genet. (2020) 11:208. doi: 10.3389/fgene.2020.00208, PMID: 32231684 PMC7083077

[39] Cruz LA Cooke Bailey JN Crawford DC . Importance of diversity in precision medicine: generalizability of genetic associations across ancestry groups toward better identification of disease susceptibility variants. Annu Rev BioMed Data Sci. (2023) 6:339–56. doi: 10.1146/annurev-biodatasci-122220-113250, PMID: 37196357 PMC10720270

[40] Chaudhary R Likidlilid A Peerapatdit T Tresukosol D Srisuma S Ratanamaneechat S . Apolipoprotein E gene polymorphism: effects on plasma lipids and risk of type 2 diabetes and coronary artery disease. Cardiovasc Diabetol. (2012) 11:36. doi: 10.1186/1475-2840-11-36, PMID: 22520940 PMC3372424

[41] Basavaraju P Moorthi PV Meyyazhagan A Devaraj I Babu K Panza E . Effects of APOE isoforms in diabetic nephropathy patients of South India. Acta Diabetol. (2025) 62:487–97. doi: 10.1007/s00592-024-02374-2, PMID: 39417844 PMC12055913

[42] Bekenova N Aitkaliyev A Vochshenkova T Kassiyeva B Benberin V . Cardiac autonomic neuropathy is not associated with apolipoprotein E gene isoforms in the Kazakh population: A case-control study. Diagnostics (Basel). (2024) 14:1978. doi: 10.3390/diagnostics14171978, PMID: 39272762 PMC11394646

[43] Shi J Cheng Z Qiu S Cui H Gu Y Zhao Q . ϵ2 allele and ϵ2-involved genotypes (ϵ2/ϵ2, ϵ2/ϵ3, and ϵ2/ϵ4) may confer the association of APOE genetic polymorphism with risks of nephropathy in type 2 diabetes: a meta-analysis. Lipids Health Dis. (2020) 19:136. doi: 10.1186/s12944-020-01307-6, PMID: 32534589 PMC7293775

[44] Tang Y Li YM Zhang M Chen YQ Sun Q . ϵ3/4 genotype of the apolipoprotein E is associated with higher risk of Alzheimer’s disease in patients with type 2 diabetes mellitus. Gene. (2019) 703:65–70. doi: 10.1016/j.gene.2019.03.024, PMID: 30890475

[45] Zeng Y Wen S Huan L Xiong L Zhong B Wang P . Association of *ApoE* gene polymorphisms with serum lipid levels and the risk of type 2 diabetes mellitus in the Chinese Han population of central China. PeerJ. (2023) 11:e15226. doi: 10.7717/peerj.15226, PMID: 37123009 PMC10135405

[46] Tsuzuki S Murano T Watanabe H Itoh Y Miyashita Y Shirai K . The examination of apoE phenotypes in diabetic patients with peripheral neuropathy. Rinsho Byori. (1998) 46:829–33., PMID: 9760837

[47] Bedlack RS Edelman D Gibbs JW 3rd Kelling D Strittmatter W Saunders AM . APOE genotype is a risk factor for neuropathy severity in diabetic patients. Neurology. (2003) 60:1022–4. doi: 10.1212/01.wnl.0000056689.50682.94, PMID: 12654974

[48] Monastiriotis C Papanas N Trypsianis G Karanikola K Veletza S Maltezos E . The ϵ4 allele of the APOE gene is associated with more severe peripheral neuropathy in type 2 diabetic patients. Angiology. (2013) 64:451–5. doi: 10.1177/0003319712453645, PMID: 22826377

[49] Chou Y Sun Z Wang Y Wang Y Ma J Zhang D . Genetic polymorphisms of apolipoprotein E in nonarteritic anterior ischemic optic neuropathy. Graefes Arch Clin Exp Ophthalmol. (2022) 260:2717–26. doi: 10.1007/s00417-022-05616-7, PMID: 35258716

[50] Liu S Liu J Weng R Gu X Zhong Z . Apolipoprotein E gene polymorphism and the risk of cardiovascular disease and type 2 diabetes. BMC Cardiovasc Disord. (2019) 19:213. doi: 10.1186/s12872-019-1194-0, PMID: 31521122 PMC6744677

[51] Zhou Z Hoke A Cornblath DR Griffin JW Polydefkis M . APOE epsilon4 is not a susceptibility gene in idiopathic or diabetic sensory neuropathy. Neurology. (2005) 64:139–41. doi: 10.1212/01.WNL.0000148587.97690.4E, PMID: 15642920

[52] Dunk MM Driscoll I Espeland MA Hayden KM Liu S Nassir R . Relationships between APOE, type 2 diabetes, and cardiovascular disease in postmenopausal women. J Gerontol A Biol Sci Med Sci. (2025) 80(2):glae246. doi: 10.1093/gerona/glae246, PMID: 39364911 PMC11775828

[53] Voron’ko OE Yakunina N Strokov IA Lavrova IN Nosikov VV . Association of polymorphic markers of the lipid metabolism genes with diabetic neuropathy in type 1 diabetes mellitus. Mol Biol. (2005) 39:206–9., PMID: 15856946

[54] Kasznicki J Kosmalski M Sliwinska A Mrowicka M Stanczyk M Majsterek I . Evaluation of oxidative stress markers in pathogenesis of diabetic neuropathy. Mol Biol Rep. (2012) 39:8669–78. doi: 10.1007/s11033-012-1722-9, PMID: 22718504 PMC3404273

[55] Bennet AM Di Angelantonio E Ye Z Wensley F Dahlin A Ahlbom A . Association of apolipoprotein E genotypes with lipid levels and coronary risk. JAMA. (2007) 298:1300–11. doi: 10.1001/jama.298.11.1300, PMID: 17878422

[56] Zhang L He S Li Z Gan X Li S Cheng X . Apolipoprotein E polymorphisms contribute to statin response in Chinese ASCVD patients with dyslipidemia. Lipids Health Dis. (2019) 18:129. doi: 10.1186/s12944-019-1069-5, PMID: 31153375 PMC6545221

[57] Koren-Iton A Salomon-Zimri S Smolar A Shavit-Stein E Dori A Chapman J . Central and peripheral mechanisms in ApoE4-driven diabetic pathology. Int J Mol Sci. (2020) 21:1289. doi: 10.3390/ijms21041289, PMID: 32075060 PMC7072920

[58] Pires M Rego AC . Apoe4 and Alzheimer’s Disease pathogenesis-mitochondrial deregulation and targeted therapeutic strategies. Int J Mol Sci. (2023) 24:778. doi: 10.3390/ijms24010778, PMID: 36614219 PMC9821307

[59] Rajič Bumber J Rački V Mežnarić S Pelčić G Mršić-Pelčić J . Clinical significance of APOE4 genotyping: potential for personalized therapy and early diagnosis of Alzheimer’s Disease. J Clin Med. (2025) 14:6047. doi: 10.3390/jcm14176047, PMID: 40943807 PMC12429504

[60] Karimi MA Vaezi A Ansari A Archin I Dadgar K Rasouli A . Lipid variability and risk of microvascular complications in patients with diabetes: a systematic review and meta-analysis. BMC Endocr Disord. (2024) 24:4. doi: 10.1186/s12902-023-01526-9, PMID: 38167035 PMC10759662

[61] Carmichael J Fadavi H Ishibashi F Shore AC Tavakoli M . Advances in screening, early diagnosis and accurate staging of diabetic neuropathy. Front Endocrinol (Lausanne). (2021) 12:671257. doi: 10.3389/fendo.2021.671257, PMID: 34122344 PMC8188984

[62] Elafros MA Andersen H Bennett DL Savelieff MG Viswanathan V Callaghan BC . Towards prevention of diabetic peripheral neuropathy: clinical presentation, pathogenesis, and new treatments. Lancet Neurol. (2022) 21:922–36. doi: 10.1016/S1474-4422(22)00188-0, PMID: 36115364 PMC10112836

[63] Theofilis P Sagris M Oikonomou E Antonopoulos AS Siasos G Tsioufis C . Inflammatory mechanisms contributing to endothelial dysfunction. Biomedicines. (2021) 9:781. doi: 10.3390/biomedicines9070781, PMID: 34356845 PMC8301477

