# Supplementary figures and images for: Apolipoprotein E genotypes are associated with diabetic peripheral neuropathy in Lebanese adults with type 2 diabetes: a case-control study
Source: Front Endocrinol (Lausanne). 2025 Dec 19;16:1738873. doi: 10.3389/fendo.2025.1738873 (PMC12757874; doi:10.3389/fendo.2025.1738873)

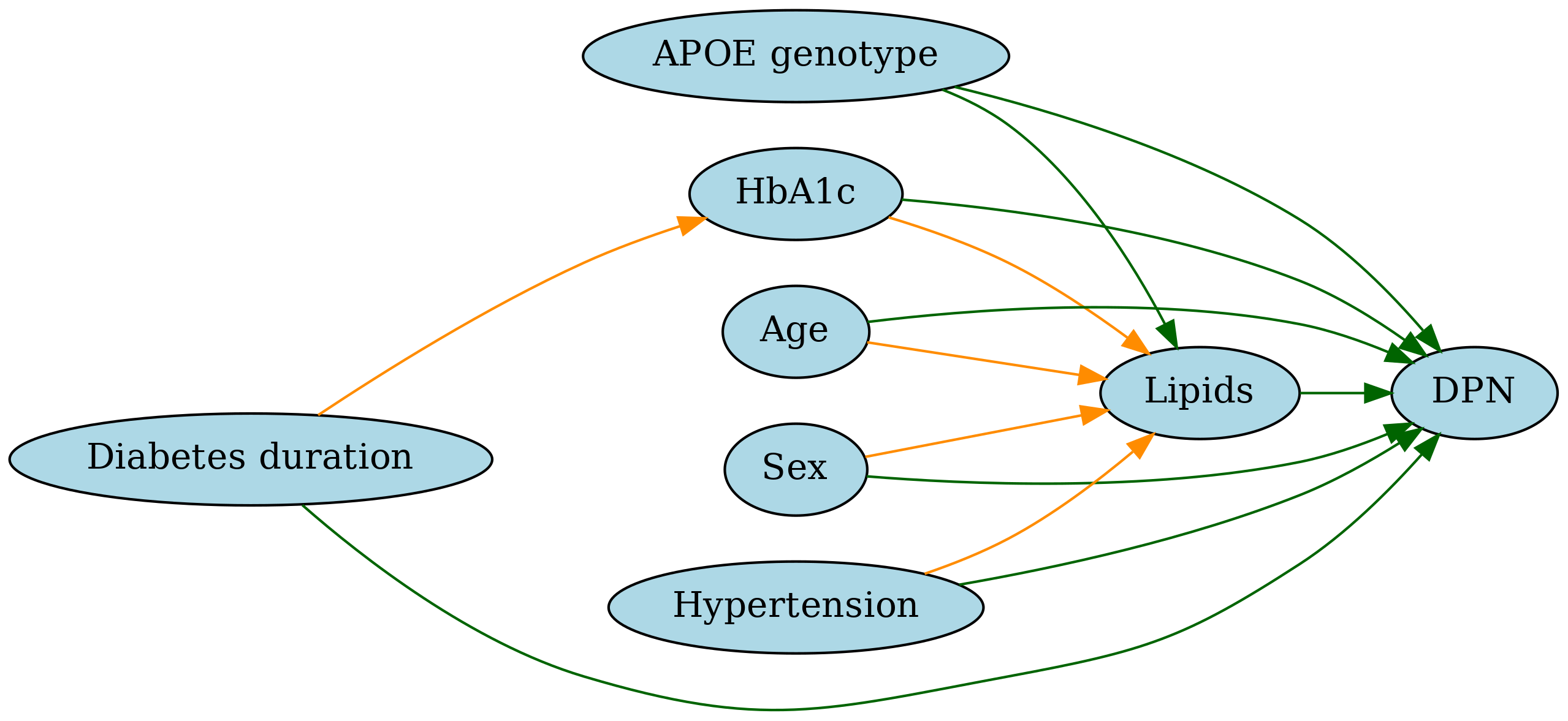

Supplement: Supplementary Figure 1 — Directed acyclic graph (DAG) illustrating the hypothesized causal structure for the association between APOE genotype and diabetic peripheral neuropathy (DPN). [file Image1.png]
