# Supplementary material for: Apolipoprotein E genotypes are associated with diabetic peripheral neuropathy in Lebanese adults with type 2 diabetes: a case-control study
Source: Front Endocrinol (Lausanne). 2025 Dec 19;16:1738873. doi: 10.3389/fendo.2025.1738873 (PMC12757874; doi:10.3389/fendo.2025.1738873)
Supplement: Supplementary file 2 [file DataSheet1.docx]

# Supplementary Note 1

# Mechanistic Basis Linking APOE Variants to Diabetic Peripheral Neuropathy (DPN)

This Supplementary Note provides a concise mechanistic summary of how APOE isoforms influence diabetic peripheral neuropathy (DPN) through lipid metabolism, oxidative stress, inflammation, microvascular dysfunction, and neuronal repair pathways.

**1. *APOE* Isoforms and Receptor Interactions**

*APOE* exists as *ε2*, *ε3*, and *ε4* isoforms, generated by two SNPs that alter amino acids 112 and 158. These substitutions modify receptor-binding affinity (LDLR, LRP1, ApoER2, HSPGs) and lipoprotein association. ε3 is metabolically neutral. ε4 alters protein folding and increases LDL receptor affinity, whereas ε2 shows markedly reduced LDL receptor binding, predisposing to remnant accumulation.

**2. Lipid Dysregulation and Neural Vulnerability**

ε4 produces an atherogenic lipid pattern (increased LDL and TG, reduced HDL), promoting endothelial dysfunction and reduced vasa nervorum perfusion. ε2 carriers may develop context‑dependent hypertriglyceridemia and remnant lipoprotein accumulation, contributing to microvascular injury. ε3 maintains balanced lipid homeostasis.

**3. Oxidative Stress and Mitochondrial Dysfunction**

ε4 shows diminished antioxidant capacity, leading to increased ROS, mitochondrial membrane depolarization, ATP depletion, and lipid peroxidation. Diabetes further exacerbates oxidative stress via AGE formation and activation of the polyol/PKC pathway. Mitochondrial dysfunction compromises axonal energy supply, Schwann cell survival, and nerve conduction.

**4. Neuroinflammation**

ApoE modulates macrophage and microglial activity. ε4 promotes a pro‑inflammatory cytokine profile (upregulated TNF‑α, IL‑1β, IL‑6; downregulated IL‑10), impairing Schwann cell function, myelin maintenance, and axonal repair. ε2 impairs neuronal repair signaling despite having a less inflammatory baseline profile. Diabetes amplifies systemic inflammation and synergizes with APOE-driven immune imbalance.

**5. Microvascular Dysfunction**

Both ε2 and ε4 contribute to endothelial injury, but through distinct mechanisms. ε4 increases oxidative burden, reduces nitric oxide, and accelerates vascular stiffness and capillary rarefaction. ε2’s remnant accumulation thickens capillary basement membranes and disrupts endothelial repair. Consequences include reduced perfusion, hypoxia, blood–nerve barrier leakage, and increased exposure of nerves to inflammatory mediators.

**6. Neuronal Maintenance and Axonal Transport**

Peripheral nerves rely on intact lipid delivery, mitochondrial energy, and neurotrophic signaling. ε4 compromises ABCA1-mediated cholesterol efflux and lipid delivery to Schwann cells, accelerating demyelination. ε4 also increases tau phosphorylation and microtubule instability, slowing axonal transport and worsening length‑dependent neuropathy. ε4 diminishes NGF/BDNF signaling, reducing regenerative capacity.

**7. Gene–Environment Interactions**

The ε4 × hypertriglyceridemia synergy (“double‑hit” model) reflects compounding metabolic and genetic stressors: dyslipidemia, inflammation, and endothelial injury converge to accelerate neural degeneration. Ethnic lipid backgrounds and genetic architecture may modulate the penetrance of *APOE* effects.

**8. Integrated Multi‑Hit Pathway**

Across isoforms, DPN arises from convergent mechanisms: systemic dyslipidemia, oxidative stress, inflammation, microvascular insufficiency, and impaired neuronal maintenance. ε4 amplifies pathogenic hits at every level; ε2 drives remnant‑associated vascular dysfunction; ε3 remains protective or neutral.

**Conclusion**

*APOE* isoforms influence susceptibility to DPN by modulating lipid homeostasis, inflammatory signaling, mitochondrial resilience, and microvascular–neuronal crosstalk. These integrated pathways provide biological plausibility for observed genotype-specific associations and support inclusion of APOE in mechanistic and precision‑risk models for DPN.
