# Supplementary material for: Apolipoprotein E genotypes are associated with diabetic peripheral neuropathy in Lebanese adults with type 2 diabetes: a case-control study
Source: Front Endocrinol (Lausanne). 2025 Dec 19;16:1738873. doi: 10.3389/fendo.2025.1738873 (PMC12757874; doi:10.3389/fendo.2025.1738873)
Supplement: Supplementary file 4 [file Table2.docx]

**Supplementary Table 2**

Global Distribution of ApoE Alleles

| **Region** | **Population/region or country** | **ε2 (%)** | **ε3 (%)** | **ε4 (%)** | **Reference** | **Notes** |
| --- | --- | --- | --- | --- | --- | --- |
|  | Lebanon | 2.6 | 88.4 | 9.0 | This study |  |
| Europe | Eastern Europe | 3–6 | 75–85 | 10–20 | [1 – 2] | General population cohorts |
|  | Western Europe | 5–8 | 78–86 | 8–14 | [1 – 2] | General population cohorts |
| Americas | North Americans | 5–8 | 77-85 | 9-15 | [3] | Mixed European ancestry cohorts |
|  | South Americans | 3–6 | 80–86 | 8–14 | [4] | Admixed urban samples |
| Asia | East Asians | 6-10 | 80-88 | 5-10 | [5] | Multiple healthy cohorts |
|  | Central Asians | 3-7 | 80-87 | 7-13 | [6] | Limited country data |
| Africa | Sub-Saharan Africans | 2-6 | 65-80 | 15-25 | [6] |  |
|  | Nigeria | 2 – 5 | 68 – 78 | 17 – 25 | [18, 19] | Community/control cohorts |
|  | South Africa | 2 – 5 | 70 – 80 | 15 – 25 | [20 – 22] | Rural community cohorts |
| Middle East | Overall | 3-7 | 78-86 | 8-15 | [6] | Aggregated healthy/general cohorts |
|  | Iran | 3-6 | 80-86 | 8-14 | [9 – 11] |  |
|  | Saudi Arabia | 3-6 | 80-86 | 8-14 | [12 – 15] | General-population/ city-based cohorts |
|  | Egypt | 3 – 6 | 78 – 85 | 9 – 15 | [16, 17] |  |
|  | Jordan | 3 – 6 | 80 – 86 | 8 – 14 | [7] | General-population data |
|  | Turkey (Marmara Region) | 3 – 6 | 80 – 86 | 8 – 14 | [8] | Regional community samples |

**References:**

1. Kolbe D, da Silva NA, Dose J, Torres GG, Caliebe A, Krause‑Kyora B, Nebel A (2023) Current allele distribution of the human longevity gene APOE in Europe can mainly be explained by ancient admixture. Aging Cell 22:e13819. doi:10.1111/acel.13819

2. Egert S, Rimbach G, Huebbe P (2012) ApoE genotype: from geographic distribution to function and responsiveness to dietary factors. Proc Nutr Soc 71:410–424. doi:10.1017/S0029665112000229

3. Belloy ME, Andrews SJ, Le Guen Y, et al. (2023) APOE genotype and Alzheimer disease risk across age, sex, and population ancestry. JAMA Neurol 80:1284–1294. doi:10.1001/jamaneurol.2023.3599

4. Arango Viana JC, Valencia AV, Páez AL, Montoya Gómez N, Palacio C, Arbeláez MP, Bedoya Berrío G, García Valencia J (2010) Prevalence of variants in the apolipoprotein E (APOE) gene in a general population of adults from an urban area of Medellín (Antioquia). Biomédica 30:140–149. doi:10.7705/biomedica.v30i2.246

5. Miyashita A, Kikuchi M, Hara N, Ikeuchi T (2022) Genetics of Alzheimer’s disease: an East Asian perspective. J Hum Genet 67:475–489. doi:10.1038/s10038-022-01050-z

6. Abondio P, Sazzini M, Garagnani P, Boattini A, Monti D, Franceschi C, Luiselli D, Giuliani C (2019) The genetic variability of APOE in different human populations and its implications for longevity. Genes 10:222. doi:10.3390/genes10030222

7. Khabour OF, Abdelhalim ES (2018) Distribution of APOE gene variations in the Jordanian population: association with longevity. J King Saud Univ Sci 32:518–522. doi:10.1016/j.jksus.2018.08.004

8. Ismail AB, Dundar MS, Erguzeloglu CO, Ergoren MC, Alemdar A, Ozemri Sag S, Temel SG (2024) Alzheimer disease–associated loci: APOE single nucleotide polymorphisms in Marmara Region. Biomedicines 12:968. doi:10.3390/biomedicines12050968

9. Iranifar E, Hamzehloie T (2016) The study of apolipoprotein E4 allele distribution in parents of Down’s syndrome children as a risk factor in Khorasan Razavi Province, Iran. Open J Genet 6:87–95. doi:10.4236/ojgen.2016.64010

10. Shahsavar F, Sabooteh T, Jafarzadeh M (2014) Distribution of ApoE polymorphisms in the Lur population. Yafteh 15(5):55.

11. Abyadeh M, Djafarian K, Heydarinejad F, Alizadeh S, Shab‑Bidar S (2019) Association between apolipoprotein E gene polymorphism and Alzheimer’s disease in an Iranian population: a meta‑analysis. J Mol Neurosci 69:557–562. doi:10.1007/s12031-019-01381-1

12. Al‑Khedhairy AA (2005) Apolipoprotein E polymorphism in Saudis. Mol Biol Rep 31:257–260. doi:10.1007/s11033-005-2713-x

13. Awad NS, El‑Tarras AS (2011) Analysis of the APO B R3500Q mutation and APOE polymorphism in Taif Saudi population using PCR‑reverse hybridization. J Mol Biomark Diagn 2:1000109. doi:10.4172/2155-9929.1000109

14. Kondkar AA, Sultan T, Azad TA, et al. (2024) Common variants rs429358 and rs7412 in APOE gene are not associated with POAG in a Saudi cohort. Biology 13:62. doi:10.3390/biology13010062

15. Almigbal TH, Batais MA, Hasanato RM, Alharbi FK, Khan IA, Alharbi KK (2018) Role of apolipoprotein E gene polymorphism in the risk of familial hypercholesterolemia: a case–control study. Acta Biochim Pol 65:415–420. doi:10.18388/abp.2017_2344

16. Arafa S, Abdelsalam S, El‑Gilany AH, Mosaad YM, Abdel‑Ghaffar A (2018) Endothelial nitric oxide synthase Glu298Asp (G894T) and apolipoprotein E gene polymorphism as possible risk factors for coronary heart disease among Egyptians. Egypt Heart J 70:393–401. doi:10.1016/j.ehj.2018.05.003

17. Khedr EM, William MB, Elhosseiny AA, et al. (2025) APOE genetic variability in an Egyptian cohort of Parkinson’s disease. Front Neurosci 19:1579968. doi:10.3389/fnins.2025.1579968

18. Bamidele OT, Olayanju O, Emuze M, et al. (2022) Allele frequencies of apolipoprotein E in a Southwestern Nigerian population on HAART. Babcock Univ Med J 5:142–149. doi:10.38029/babcockunivmedj.v5i2.160

19. Okubadejo NU, Okunoye O, Ojo OO, et al. (2022) APOE E4 is associated with impaired self‑declared cognition but not disease risk or age of onset in Nigerians with Parkinson’s disease. npj Parkinson’s Disease 8:55. doi:10.1038/s41531-022-00411-x

20. Soo CC, Farrell MT, Tollman S, Berkman L, Nebel A, Ramsay M (2021) Apolipoprotein E genetic variation and its association with cognitive function in rural‑dwelling older South Africans. Front Genet 12:689756. doi:10.3389/fgene.2021.689756

21. Masemola ML, Alberts M, Urdal P (2007) Apolipoprotein E genotypes and their relation to lipid levels in a rural South African population. Scand J Public Health 35(Suppl 69):60–65. doi:10.1080/14034950701355635

22. Tanyanyiwa DM, Marais AD, Byrnes P, Jones S (2016) The influence of ApoE genotype on lipid profile and lipoproteins during normal pregnancy in a Southern African population. Afr Health Sci 16:1123–1132. doi:10.4314/ahs.v16i4.10.
